# An extract of the medicinal mushroom *Agaricus blazei *Murill can protect against allergy

**DOI:** 10.1186/1476-7961-7-6

**Published:** 2009-05-05

**Authors:** Linda K Ellertsen, Geir Hetland

**Affiliations:** 1 Department of Environmental Immunology, Norwegian Institute of Public Health, Oslo, Norway; 2 Department of Immunology and Transfusion Medicine, Oslo University Hospital, Ulleval, Oslo, Norway

## Abstract

**Background:**

*Agaricus blazei *Murill (AbM) is an edible Brazilian mushroom that has been used in traditional medicine for a range of diseases. It has been shown to have anti-infection and anti-tumor properties in the mouse, which are due to induction of Th1 responses. On the other hand, IgE-mediated allergy is induced by a Th2 response.

**Objective:**

Since according to the Th1/Th2 paradigm an increased Th1 response may promote a reduced Th2 response, the aim was to examine whether AbM had anti-allergy effects.

**Methods:**

A mouse model for allergy was employed, in which the mice were immunized s.c. with the model allergen ovalbumin (OVA). Additionally, the animals were given a mushroom extract, AndoSan™, mainly (82%) containing AbM, but also *Hericium erinaceum *(15%) and *Grifola frondosa *(3%), or PBS p.o. either a day before or 19 days after the immunization. The mice were sacrificed on day 26, and anti-OVA IgE (Th2 response) and IgG2a (Th1 response) antibodies were examined in serum and Th1, Th2 and Treg cytokines in spleen cells cultures.

**Results:**

It was found that the AndoSan™ extract both when given either before or after OVA immunization reduced the levels of anti-OVA IgE, but not IgG2a, in the mice. There was a tendency to reduced Th2 relative to Th1 cytokine levels in the AndoSan™ groups.

**Conclusion:**

This particular AbM extract may both prevent allergy development and be used as a therapeutical substance against established allergy.

## Background

*Agaricus blazei *Murill (AbM) of the family *Basidiomycetes *is a popular edible medicinal mushroom, originally native to a small village, Piedade, in the highland areas of Atlantic forest near São Paulo, Brazil. It has traditionally been used for the prevention of a range of diseases, including cancer, hepatitis, atherosclerosis, hypercholesterolemia, diabetes and dermatitis [1,2]. Because of its alleged health effects, the mushroom was brought to Japan in the mid-60-ies and subjected to biomedical research. AbM was found to be rich in immuno-modulating substances such as β-glucans [3,4] and proteoglycans [5], and it had anti-infection [6,7] and anti-tumor [4,5] effects in mice.

Anti-tumor and anti-infection immunity are both due to Th1 responses, which also do promote autoimmune disease when overshooting. On the other hand, anti-helminth and anti-rejection immunity are due to Th2 responses, which may also induce IgE-mediated allergy, whereas delayed-type hypersensitivity is believed to involve Th1 cells. Since, according to the original Th1/Th2 dichotomy [8] there is an inverse relationship between Th1 and Th2 responses, we set out to look for substances that increased Th1 responses and thus, presumably, would reduce allergy. Moreover, we looked for substances with broad immunogenic specificity and hence a broad range of possible therapeutical activity. This criterion fits substances containing so-called pathogen-associated molecular patterns, which stimulate innate immunity via binding to a few different receptors with broad specificities like Toll-like receptors and dectin-1.

In order to test putative functional Th1-stimulating substances, a mouse model for systemic bacterial infection was chosen rather than a tumor model, because of the more rapid outcome of an anti-bacterial than an anti-tumor response. We tested different β-glucans, which are known stimulators of innate immunity with anti-tumor [9] and anti-infection [10] activities. We found that one β-1,3-glucan from *Sclerotinia sclerotiorum *was highly protective against sepsis in a mouse model for systemic *S. pneumoniae*, although only when given i.p. and not p.o. [11]. However, surprisingly, we detected that s.c. administration of both this β-glucan and other β-glucans from barley and baker's yeast, in addition to moulds *per se*, also increased specific IgE levels in a mouse model for allergy [12,13]. This is in agreement with the finding of increased allergic responses of mold-derived β-1,3-glucan in an airway inhalation model in the mouse [14]. Since AbM is another more recently discovered source of strong innate stimulatory properties [15,16], with a high content of β-glucan and anti-tumor properties in the mouse [3], we tested whether extracts of AbM from different producers had anti-infection effects in the said mouse model for pneumococcal sepsis. We found that the current extract, AndoSan™, containing approximately 80% of AbM and 20% of two other *Basidiomycetes *mushrooms; *Hericium erinaceum *and *Grifola frondosa*, was the most effective: It was the only extract that decreased bacteremia statistically significantly and increased the survival rate of the exposed animals [17]. Moreover, it had more profound anti-infection effect even when given p.o. via a gastric catheter than did any of the above β-glucans given i.p..

There are anecdotes about persons who have used AbM for other purposes than allergy, and who have experienced less allergic symptoms when ingesting the remedy. To our knowledge the very few papers on AbM or other *Basidiomycetes *mushrooms and allergy in English scientific literature rather report on induction of allergy; cheilitis and increased delayed-type sensitivity due to AbM [18,19], hypersensitivity pneumonitis caused by *Grifola frondosa *[20], and allergic contact dermatitis from *Hericeum erinaceum *exposure [21]. Based on preliminary anti-infection and anti-allergy results in our laboratory with the current extract of mainly AbM (AndoSan™), a patent application was filed in 2004 [22]. There are other publications on beneficial effects of the mushrooms in the patent literature, foremost of Japanese origin: One patent (A61K 35/84, 05.08.2002) claims that an essence extracted from mycelium of *Basidiomycetes*, including *Hericium erinaceum*, can prevent and cure allergic symptoms, especially atopic dermatitis. Another (WO 02/15917) claims the use of AbM in treatment of autoimmune and skin diseases, due to down-regulation of immune function. Yet another (WO 93/207923) describes the isolation from *Agaricus hortensis *of anti-allergic components, especially for dermatological usage. Extracts of AbM have also been found to have anti-allergic effect based on inhibition of basophilic leukocytes (US2003/0104006). Finally, EP0413053 describes a process for producing an anti-allergic substance from *Basidiomycetes *mycelium, including that of AbM and *Grifola frondosa*.

The aim of the present study was to examine whether the extract that was most effective against systemic pneumococcal infection, also could protect against allergy development when given to a mouse model for allergy. For this purpose the model allergen ovalbumin (OVA) was injected s.c. and AbM extract as adjuvant was given orally, and levels of specific IgE and IgG2a antibodies were determined in serum. In addition, Th1, Th2 and Treg cytokines were measured in supernatants of cultured spleen cells from the mice.

## Methods

### Mice

These were inbred, female, pathogen-free, 6–8 weeks old NIH/OlaHsd, C57Bl/6 and Balb/c obtained from Gl. Bomholt gård Ltd (Ry, Denmark) and rested for 1 week after arrival. They were housed 8 animals per cage, individually earmarked, and given water and egg-free feed ad libitum. Experiments were performed according to law and regulations for animal experiments in Norway, which are in agreement with the Helsinki declaration, and they were approved by the local Animal Board under the minister of Agriculture in Norway.

### Reagents

An aqueous extract of mycelium of AbM (82%), containing additionally *Hericium erinaceum *(15%) and *Grifola frondosa *(3%) (AndoSan™), grown commercially, was given by ACE Co., Ltd., Gifu, Japan. It was stored at 4°C in dark bottles and kept sterile until being instilled intragastrically in the mice. The AbM mixed powder contains per 100 g the following constituents: moisture 5.8 g, protein 2.6 g, fat 0.3 g, carbohydrates 89.4 g of which β-glucan constitutes 2.8 g, and ash 1.9 g, and its final concentration was 340 g/l. The amount per liter of the extract for sodium was 11 mg, phosphorus 254 mg, calcium 35 mg, potassium 483 mg, magnesium 99 mg and zinc 60 mg. The LPS content of AndoSan™ was found, using the *Limulus *amebocyte lysate test (COAMATIC Chromo-LAL; Chromogenix, Falmouth, MA, USA) with detection limit 0.005 EU/ml (1 EU = 0.1 ng/ml), to be a miniscule concentration of <0.5 pg/ml. The results from tests for heavy metals were conformable with strict Japanese regulations for health foods. AndoSan™ had been heat-sterilized (124°C for 1 h) by the producer. Since this mushroom extract is a commercial product, the method for its production is a business secret. Ovalbumin (OVA) (Sigma, St. Louis, MO, USA; cat.no. A7641) and Al(OH)_3 _were dissolved in PBS of pH 7.3, and each animal was immunized with 10 μg of OVA and 2 mg of Al(OH)_3 _in a total volume of 0.5 ml in the tail base.

### Experimental design

Groups of 8 mice were given either 200 μl (according to their assumed maximal ventricular volume) of the AbM extract, AndoSan™, or PBS orally via a gastric tube and injected a day later with OVA +Al(OH)_3 _s.c. in the tail base or injected first with OVA +Al(OH)_3 _s.c. and given AndoSan™ or PBS p.o. on day 19. With Balb/c mice both OVA and 20 μl of AndoSan™ or PBS were injected s.c. in one hind foot pad (for Balb/c mice). Then both groups were boosted with OVA s.c. on day 20, before sacrifice and exanguination and removal of the spleen or the foot pad-draining popliteal lymph nodes (PLN) (for Balb/c mice), on day 26. Some mice (C57Bl/6) were given additional AbM or PBS treatment on both day -1 and day 19 before the OVA boosting. The scheme in Table 1 shows the different set-ups.

**Table 1:** Scheme for experimental design in murine allergy model

Exp #	# Mice, strain	Treatment before and/or after OVA immunization			Harvest
1	16 NIH/OlaHsd	AndoSan™ or PBS p.o.* (200 μl) ↓			serum, spleen ↑

2	16 NIH/OlaHsd			AndoSan™/PBS p.o. ↓	serum, spleen ↑

3	8 C57Bl/6	AndoSan™/PBS p.o. and/or ↓		AndoSan™/PBS p.o. ↓	serum, spleen ↑

4	8 Balb/c	AndoSan™/PBS s.c. in foot pad (20 μl)** ↓			serum, PLN ↑

Day	-1	0	19,	20	26

Immunization		↑ OVA (10 μg) + Al(OH)3 s.c. in tailbase or foot pad +		↑ OVA s.c	↑ Sacrifice

*200 μl of AndoSan™ or PBS was given p.o. via a gastric catheter. ** OVA and Al(OH)_3 _(2 mg) was dissolved in AndoSan™ or PBS before s.c. injection in foot pad.

### Spleen cell cultures

The spleen was removed from each sacrificed mouse and put in a tube containing Hank's Balanced Salt Solution (HBSS; Gibco BRL, Paisley, Scotland). A single cell suspension was prepared under sterile condition by placing the spleen on top of a wire-net in a Petri dish containing 2 ml HBSS. The spleen was punctured by a canula (BD Microlance™ 3 needle, Becton Dickinson AB, Sweden) and thereafter a bended glass staff was used to rub the cells from the spleen capsule through the wire net to make a single cell suspension. The cell suspensions were washed in HBSS and resuspended in RPMI (RPMI 1640 culture medium with 20 mM L-glutamine (Gibco)), containing 10% FCS, 100 U penicillin G and 0.1 mg/ml streptomycin (PAA Laboratories GmbH). The cell concentration was measured with a Coulter Counter ZI (Beckman Coulter Inc., FL, USA). The spleen cells were seeded into a 24-well culture plates (Costar Inc., NY, USA) to a final concentration of 5 × 10^6 ^cells/ml. OVA or Con A were added to a final concentration of 1 mg/ml and 6 μg/ml, respectively, except for unstimulated controls. The cells were cultured at 37°C and in 5% CO_2 _for 48 or 72 hours. Thereafter the plates were centrifuged at 1200 rpm for 5 minutes, and supernatants were collected and stored at -80C until analysis.

### Assays

Mouse IgE anti-OVA and IgG2a anti-OVA antibodies were measured in serum, and levels of cytokines IFNγ, IL-2 (Th1 response), IL-4, IL-5 (Th2 response) and IL-10 (Treg cytokine) in cell culture supernatants by ELISAs. Whereas the former Ig ELISAs were in-house (sandwich anti-OVA IgE and simple anti-OVA IgG2a [13]) and the cut-off set to give negative results in serum from naïve mice, the ones for the cytokines were from R&D Systems, Minneapolis, MN, USA. The excised PLN from both injected and non-injected hind limb were weighed and compared as a parameter for local inflammation.

### Statistics

Sigma Stat (Systat Software, Inc., 1735 Technology Drive Suite 430 San Jose, CA) statistical and graphics package was used. When the data were normally distributed parametric assays were used, otherwise non-parametric assays. Student's t-test was used for comparing two groups. One-way ANOVA was used for single repeated measurements, and two-ways ANOVA for two experiments with repeated measurements. P values below 0.05 were considered statistically significant.

## Results

### Serum anti-OVA IgE and IgG2a antibodies

**Figure 1:**
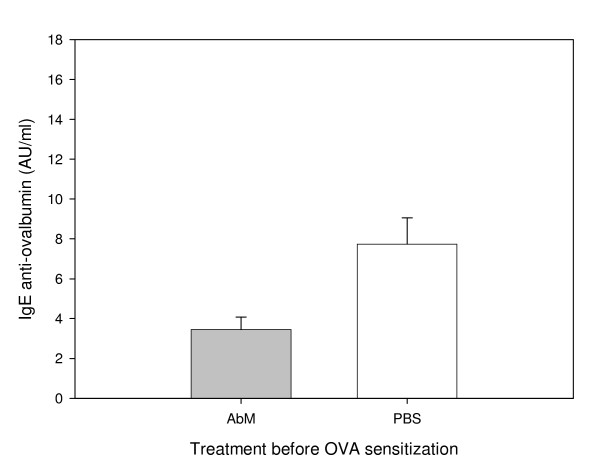
**Levels of OVA-specific IgE measured in mouse serum on day 26 after OVA-pretreatment with AbM**. Mice were given 200 μl of AndoSan™ extract or PBS intragastrically on day -1 and injected with 10 μg of OVA s.c. in the tail base on day 0 and again on day 20, before exsanguination for serum on day 26. Values are given in arbitrary units (AU)/ml and means + 1 s.e.m. for groups of 16 mice (groups of 8 per each of 2 experiments). Anti-OVA IgE levels were lower in AbM (AndoSan™) than in PBS treated groups (p = 0.002, two-way ANOVA).

**Figure 2:**
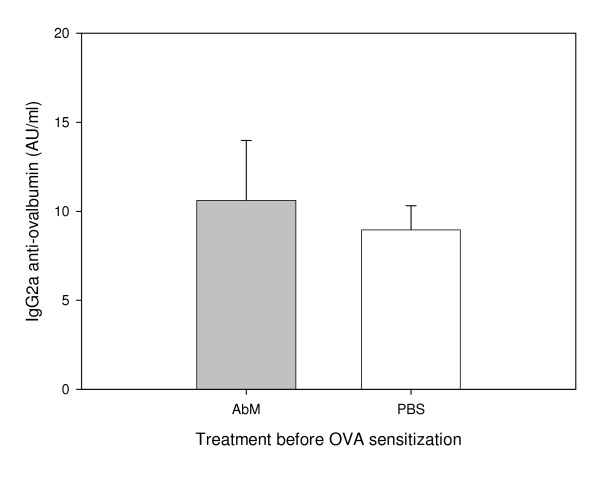
**Levels of IgG2a measured in mouse serum on day 26 after OVA-pretreatment with AbM**. Mice were given 200 μl of AndoSan™ extract or PBS intragastrically on day -1 and injected with 10 μg of OVA s.c. in the tail base on day 0 and again on day 20, before exsanguination for serum on day 26. Values are given in arbitrary units (AU)/ml and means + 1 s.e.m. for groups of 16 mice (groups of 8 per each of 2 experiments).

**Figure 3:**
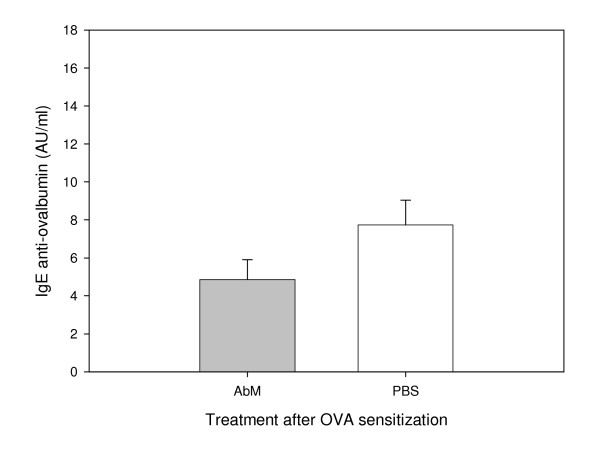
**Levels of OVA-specific IgE measured in mouse serum on day 26 after OVA-post treatment with AbM**. Mice were injected with 10 μg of OVA on day 0 and given 200 μl of AndoSan™ extract or PBS intragastrically on day 19, before OVA booster on day 20 and sacrifice on day 26. Values are given in AU/ml and means + 1 s.e.m. for groups of 16 mice (groups of 8 per each of 2 experiments). Anti-OVA IgE levels were lower in AbM (AndoSan™) than PBS treated groups (p = 0.048, two-way ANOVA).

**Figure 4:**
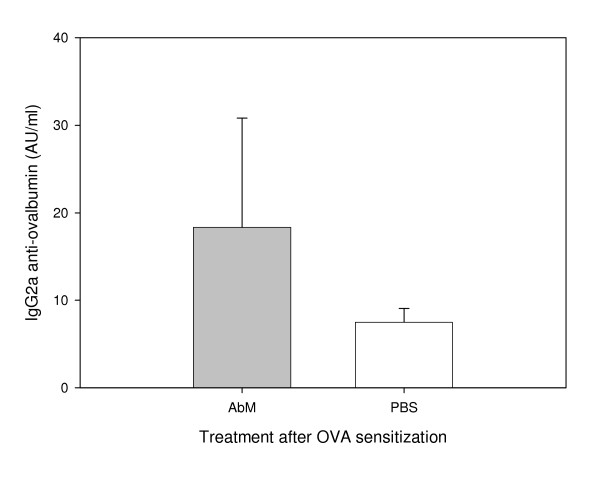
**Levels of IgG2a measured in mouse serum on day 26 after OVA-post treatment with AbM**. Mice were injected with 10 μg of OVA on day 0 and given 200 μl of AndoSan™ extract or PBS intragastrically on day 19, before OVA booster on day 20 and sacrifice on day 26. Values are given in AU/ml and means + 1 s.e.m. for groups of 16 mice (groups of 8 per each of 2 experiments).

We used a mouse model for allergy to examine whether the medicinal mushroom AbM could protect against this disease. Experiments were conducted in three mouse strains with OVA as model allergen and a mushroom extract, AndoSan™, mainly containing AbM, or PBS control as adjuvant. In two experiments with NIH/Ola mice, AbM treatment prior to OVA immunization reduced the levels of serum anti-OVA IgE antibodies significantly (p = 0.002, two-way ANOVA) compared with similar PBS pre-OVA treatment (Figure 1) when the animals were sacrificed about 4 weeks after OVA immunization. The levels of serum anti-OVA IgG2a tended to be higher in the AbM group (Figure 2), but were not statistically significantly different from the PBS control. Furthermore, when AbM, as compared with PBS, was given near 3 weeks after the allergen immunization of such mice, this treatment also significantly reduced the levels of anti-OVA IgE (p = 0.048, two-way ANOVA) (Figure 3). In these two experiments the levels of anti-OVA IgG2a in the AbM group, relative to PBS, seemed to be even higher (Figure 4) than observed above, but were due to large variation not statistically different from the control.

**Figure 5:**
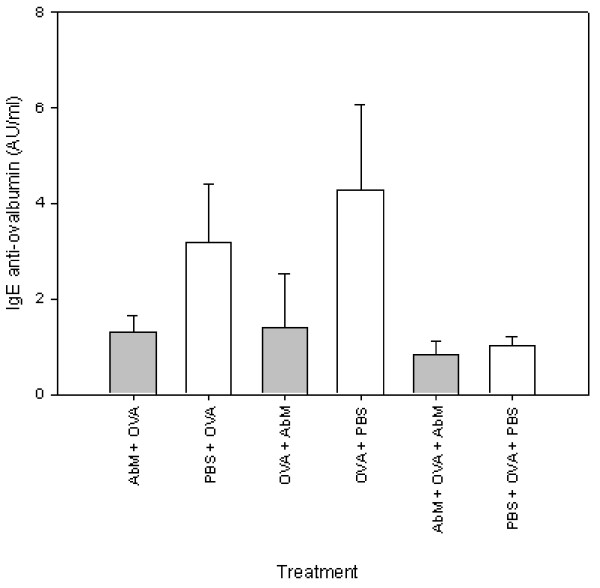
**Levels of OVA-specific IgE measured in mouse serum on day 26 after either pre- or post-OVA treatment with AbM**. Mice were either given 200 μl of AndoSan™ extract or PBS intragastrically on day -1 and injected with 10 μg of OVA s.c. in the tail base on day 0, or injected with OVA on day 0 and given AndoSan™ extract or PBS intragastrically on day 19, or given AndoSan™ extract or PBS both before (day -1) and after (day 19) OVA injection (day 0). All groups were OVA boosted on day 20 and sacrificed on day 26. Values are given in AU/ml and means + 1 s.e.m. for groups of 8 mice (p = 0.064, one-way ANOVA for difference between the groups).

The next set-up was similar to the ones above, but with C57Bl/6 mice and included groups that were treated with AbM or PBS either before or after OVA immunization, or both before and after the immunization. Figure 5 shows a tendency towards lower anti-OVA IgE levels in the AbM compared with PBS treated groups (p = 0.064, one way ANOVA), albeit the levels of specific IgE of the PBS-OVA-PBS control (last column in Figure 5) was relatively far lower than the two other PBS controls. The IgG2a levels were all-over below the detection limit of the assay and thus too low for data analysis. In a third set-up with Balb/c mice, a similar but statistically not significant trend of AbM-induced lower IgE and higher serum anti-OVA IgG2a levels was still found when using the foot pad of the mice for s.c. injection of both OVA and a 1/10 volume of AndoSan™ (data not shown).

### Cytokines in spleen cell cultures and weight of PLN

Occasionally, there were in single experiments reduced levels (p < 0.05), except increased levels once for IL-2, and otherwise no significant differences in all the five cytokines measured; IFNγ, IL-2, IL-4, IL-5 and IL-10, in spleen cell culture supernatants from animals treated with AbM relative to PBS control, either before or after OVA immunization. Table 2 gives cytokine levels as indices of those for AbM-treated relative to those for PBS treated controls. For each experiment the highest read-outs above the detection limit of each assay was used, for set-up with either OVA or Con A in vitro stimulated cell cultures. When all indices for all groups of Th2 cytokines (mean index: 0.87 ± 0.05) were compared with all indices of Th1 cytokines (mean index: 1.07 ± 0.05), Th2 cytokines were significantly lower (p = 0.026). Hence, there seemed to be a tendency of reduced Th2 relative to Th1 cytokine levels in the AbM groups. There were similar weights of the excised PLN from the AbM and PBS groups, suggesting no local inflammatory response to the mushroom extract.

**Table 2:** Cytokines in supernatants of cultured spleen cells from mice treated with AndoSan™ or PBS p.o. before or after sensitization against OVA s.c..

Cytokine	Pre-OVA treatment	Post-OVA treatment
	
	AndoSan™/PBS index	AndoSan™/PBS index
IFNγ	1.13* ± 0.06	0.90* ± 0.11

IL-2	1.06*, ** ± 0.13	1.07 ± 0.00

IL-4	0.95 ± 0.15	0.82* ± 0.10

IL-5	0.83 ± 0.08	0.78 ± 0.05

IL-10	0.88* ± 0.08	0.89 ± 0.01

The indices are given as mean ± s.e.m. of 2–5 experiments in duplicates, each comprising 8 mice per group, of the highest read-out data of either OVA or Con A stimulated cultures for either 48 h or 72 h is given for each experiment. *In one of the experiments cytokine levels in supernatants of cell cultures from AndoSan™ treated mice were lower than from PBS treated mice (p < 0.05). **In one of the experiments cytokine levels in supernatants of cell cultures from AndoSan™ treated mice were higher than from PBS treated mice (p < 0.05). All over, indices for Th2 cytokines were lower than indices for Th1 cytokines (p = 0.026). The lack of data from some experiments is due to values below detection limit of assay.

## Discussion

Our results are strengthened by the similar findings, observed in two different mouse strains after s.c. injection of OVA in the tail base, and in a third mouse strain (Balb/c) after s.c. injection of both mushroom extract and OVA in the foot pad. In the latter Th2-prone mice the so-called PLN assay was used, which was originally employed for toxicological screening of substances that would inflame the foot pad-draining PLN, but which is also convenient for examining systemic IgE response in serum to an allergen given with adjuvant [23]. The lacking increase in PLN weight in mice injected AbM extract relative to PBS in the foot pad, agrees with the assumed anti-inflammatory anti-allergic effect of the AbM as seen from the tendency of generally lowering of Th2 cytokine levels in spleen cell cultures ex vivo.

Increased specific IgE levels are not equivalent with allergic disease, but a prerequisite for IgE-mediated allergy. Hence, our findings of decreased anti-OVA IgE levels secondary to AbM intake in animals that were otherwise sensitized to OVA, strongly indicates a protective effect of AbM against IgE-mediated allergy. We did not examine allergy signs in the mice. These would have been similar to egg allergy, as in food allergy. Possible skin rashes would have been difficult to assess in the mice, and nude mice could not have been used because they lack normal lymphocytes, which are a prerequisite for an allergic immune response. In possible follow-up studies, the allergen should be given via the natural route; e.g. p.o. if using ovalbumin, although this would be costly. Instead, a common food allergen like peanut could have been used, or if one wished to examine airways allergy in the case of aeroallergens, another cheap aeroallergen like birch pollen, although with novel ELISAs for these antigens. The finding of relatively far lower anti-OVA IgE levels in the repeated PBS controls in Figure 5, may be due to the stress invoked by such repeated intragastric procedure. In preliminary experiments, in which repeated pre-OVA treatment of mice with the mushroom extract or PBS was delivered intragastrically by the highly trained technicians to increase the dose, all mice looked sick and one animal died, presumably from stress, which is known to impair immunity.

Previously, we have used pure β-glucans from yeast and fungi together with ovalbumin s.c. in the very same PLN model and, contrary to the present observation, found increased specific anti-OVA IgE levels in serum [12,13]. Hence, either the administration route is critical, or the particular β-glucans of the current mushroom extract does either promote a different outcome than the other β-glucans, or other stronger anti-allergic immunomodulating substances in the mushroom extract do overcome a possible general "pro-allergic" effect of β-glucans. If the latter is true, we assume that the anti-allergy effects of the AbM extract in vivo is mediated via immunomodulating substances in the extract that are smaller and more readily absorbable than β-glucans.

As to possible side effects, there are conflicting reports regarding the effect of AbM on liver function. Whereas one report suggests that use of AbM for several weeks may have induced severe hepatic dysfunction in three cancer patients [24], another says that AbM extract normalized liver function in patients with chronic hepatitis B virus infection [25]. Moreover, our studies on patients with chronic hepatitis C virus infection [26] and on AbM intake in healthy volunteers [27], revealed no pathological effect whatsoever on hematological parameters including those for liver-, pancreatic- and renal function, even when volumes equivalent by body weight to that given to the mice, were taken.

The generally observed AbM-induced all-over reduction in Th2 cytokines IL-4 and IL-5 relative to Th1 cytokines IFNγ and IL-2 production ex vivo in our present cultures of spleen cells, agrees with the original Th1/Th2 dichotomy [8]. However, this theory has been modified towards suggesting that T regulatory cells are crucial for fine-tuning both Th1 and Th2 responses by the regulatory cytokines IL-10 and TGF-β. However, our measurement of suggestive reduced levels of the Treg cytokine IL-10 in the AbM groups, is difficult to interpret. In contrast, when the extract was given in vitro to cell cultures there was an increase in proinflammatory cytokines [15]. This apparent discrepancy must be due to the fact that whereas cells in vitro are subjected to all substances in the extract including β-glucans with large m.w., which are abundant in AbM [3], mainly smaller substances are taken up from the digestive tract in humans and are active in the blood in vivo. Although, β-glucans in the intestines could stimulate Peyer's patches in jejunum, we have in fact observed that the genes in leukocytes predominantly affected by AbM in vitro and in vivo were quite different [26,28]. Whereas genes related to proinflammatory cytokines were strongly induced in vitro – presumably by β-glucan, genes involved in cell signalling and cycling and transcriptional regulation and thus foremost related to anti-tumor defence, were upregulated in vivo [26]. Thus, the microarray analyses agree with the assumption that AbM extract especially promotes a Th1 anti-tumor and anti-infection response in the body and hence reciprocally inhibits a Th2 response. This is supported by the reported immuno-modulatory effects of AbM in mice [19].

β-glucans may stimulate macrophages and other cells of innate immunity after binding to cellular receptors like CD11b/18, Toll-like receptor and dectin-1 [reviewed in [17]]. Stimulation by AbM of peripheral blood leukocytes resulted both in an upregulation of such receptors [26,28,29], activation of NFKB via TLR2 stimulation [30], and mediation via them of increased release of proinflammatory cytokines [15] and Th1 cytokines IFNγ, IL-12, and IL-23α [16,28,31]. Although one report of reduced release of Th2 cytokine IL-4 after AbM stimulation in vitro also found reduced IL-2 and IFNγ levels [32], IL-12- and IFNγ-mediated NK cell activation by AbM p.o. has been documented in mice [16]. Even though the present AbM extract should occasionally give reduced IFNγ levels, the increased expression of the IFN receptor gene after AbM extract intake in humans [26], may overcome a reduction in the concentration of the ligand and result in an increased Th1 response. When measuring different cytokines in serum from humans after 12 days intake of the current AndoSan™ extract mainly containing AbM, there was a significant reduction in both pro-inflammatory, Th1 and Th2 cytokines [31]. This indicates a general anti-inflammatory effect of AndoSan™ in vivo, which agrees with its current anti-allergic effect.

For intragastric delivery of AbM extract a volume of 200 μl was chosen because this is, according to our veterinary, the maximal ventricular volume in a 5–6 weeks old mouse. In an initial experiment, we tried to give the AbM extract repeatedly on subsequent days via a gastric catheter in order to possibly inhibit the specific IgE response completely. However, this procedure was dropped because it was too stressful for the mice even in hands of our well-trained technicians. The unexpected result in the last two columns of Figure 5 may in fact reflect this concern. Also, we did not use a higher concentration of this extract than what was sold on the health food market. If translated to human intake, the equivalent of 200 μl to a 25 g mouse would be 560 ml to a 70 kg individual. In fact, a daily low intake in healthy volunteers of 60 ml AndoSan™ for 12 days gave a significant 50% reduction in levels of the allergy-promoting cytokine IL-4 in blood and left the other allergy-related cytokines IL-5, IL-7 and IL-13 at negligible levels [27]. Addition of AbM extract to drink water for the mice in our set-up would have been more natural, but the intake of AbM is impossible to monitor as accurately as with intragastric delivery. In the current allergy model we did not test other extracts of AbM from other manufacturers that did not have a significant effect against pneumococcal infection in mice [17]. Therefore, the question is not fully answered as to whether there is an absolute link between the anti-bacterial infection and anti-allergy effect of a substance or an extract like AndoSan™. Moreover, even though we have seen that *Agaricus *bM is the main TLR2 stimulating mushroom of AndoSan™ [30], it is likely that the former anti-bacterial and current anti-allergic effect of this mixed mushroom product may be partly due to possible synergistic effects of the other mushrooms, *Hericium erinaceum *and *Grifola frondosa*, and components thereof contained in the extract. The mouse model of allergic airways disease should be used in a follow-up study with OVA and the mushroom extract in order to confirm that also allergic symptoms like development of airways hyper responsiveness are reduced by AndoSan™ intake. Whether the AbM extract is effective against allergy in the human setting must be tested in a clinical trial, e.g. in persons with aeroallergy during the pollen season taking 60 ml a day for a few weeks.

## Conclusion

From our results with mice we conclude that a mushroom extract, mainly containing AbM, may prevent the development of IgE-mediated allergy when given before allergen immunization. Even more interesting, the extract seemed to have a therapeutic effect when given together with or as late as 3 weeks after the allergen immunization. Three weeks in the mouse equals several months in a human, suggesting that also established allergy in patients can be reverted.

## Competing interests

Possible conflict of interest: GH filed a patent application (WO2005/065063: "Use of the mushroom *Agaricus blazei *Murill for the production of medicaments suitable for treating infections and allergies") with priority Jan 2004, based on preliminary anti-infection and anti-allergy experiments in 2003. LKE has no competing interests.

## Authors' contributions

LKE supervised the animal experiments and the Ig, cytokine and other measurements that were performed together with technicians. GH had the idea for the project and did most of the data analysing and writing, and both authors collaborated on the study design.
